# Successful subclavian transcatheter aortic valve replacement in a nonagenarian patient: Case report and review of literature

**DOI:** 10.1097/MD.0000000000028702

**Published:** 2022-01-28

**Authors:** Seok Oh, Ju Han Kim, Dae Young Hyun, Kyung Hoon Cho, Min Chul Kim, Doo Sun Sim, Young Joon Hong, Youngkeun Ahn, Myung Ho Jeong, Yochun Jung, Kyo Sun Lee

**Affiliations:** a Department of Cardiology, Chonnam National University Hospital, Gwangju, Korea; b Department of Cardiology, Chonnam National University Medical School, Gwangju, Korea; c Department of Thoracic Cardiovascular Surgery, Chonnam National University Hospital, Gwangju, Korea.

**Keywords:** aortic valve stenosis, nonagenarians, transcatheter aortic valve replacement

## Abstract

**Rationale:**

In super-aged patients with severe symptomatic aortic stenosis, transcatheter aortic valve replacement (TAVR) is a good treatment option, and the number of TAVR-eligible elderly patients is expected to grow exponentially. We present the case of a nonagenarian woman with severe aortic stenosis who underwent successful subclavian TAVR.

**Patient concerns:**

A 90-year-old Korean woman was brought to our department with dyspnea. On physical examination, a grade IV systolic murmur was auscultated in both the upper sternal borders and the left lower sternal border.

**Diagnosis:**

A transthoracic echocardiogram showed heavy calcification of the aortic valve with an increase in both peak velocity (4.36 m/s) and mean pressure (44.8 mm Hg), indicating severe symptomatic aortic stenosis.

**Interventions:**

After a heart team conference involving interventional cardiologists, cardiac surgeons, and anesthesiologists, subclavian TAVR was performed. Using the left subclavian artery, we successfully deployed a self-expandable valve prosthesis (CoreValve^TM^ Evolut R^TM^, Medtronic Inc., Minneapolis, MN).

**Outcomes:**

After TAVR, transthoracic echocardiogram showed a decline in both peak velocity (2.06–2.14 m/s) and mean pressure (7.42–7.95 mm Hg) with an increase in the aortic valve area (1.12 cm^2^). The patient's dyspnea symptoms improved dramatically.

**Lessons:**

In addition to femoral TAVR, subclavian TAVR may be feasible and safe in super-aged patients.

## 1 Introduction

Aortic stenosis (AS) is a major valvular heart disease in the elderly population, characterized by aortic valve (AoV) thickening and calcification, with motion limitation of the valvular leaflets, resulting in left ventricular outflow obstruction.^[1,2]^ To date, AoV replacement has been the only curative treatment option for severe symptomatic AS.

After the revolutionary advent of transcatheter AoV replacement (TAVR) by Alain Cribier,^[3]^ it has become a well-known treatment alternative to surgical AoV replacement (SAVR) in patients with severe symptomatic AS. As some randomized controlled trials have shown that TAVR is noninferior or even superior to SAVR in these patients,^[4–9]^ the clinical indications for TAVR have been expanded to a larger pool of eligible populations.^[10]^ As the number of octogenarian or nonagenarian patients with severe symptomatic AS is likely to increase dramatically, the number of very old patients eligible for TAVR is expected to grow exponentially.^[11]^ As these patients tend to have a high surgical risk with a high burden of morbidity and mortality, many clinicians are reluctant to treat them with SAVR. In other words, TAVR can be a good treatment option for octogenarian or nonagenarian patients with AS.

Herein, we report an extremely unusual case of a nonagenarian patient with severe symptomatic AS who was successfully treated with transsubclavian TAVR (TS-TAVR). This study was conducted in accordance with the ethical standards of the Declaration of Helsinki. The patients’ clinical information and data were available in the electronic medical records of the Chonnam National University Hospital. This study was exempt from review by the Institutional Review Board (IRB) of our hospital (IRB Number: CNUH-EXP-2021–399).

## 2 Case report

**Figure 1:**
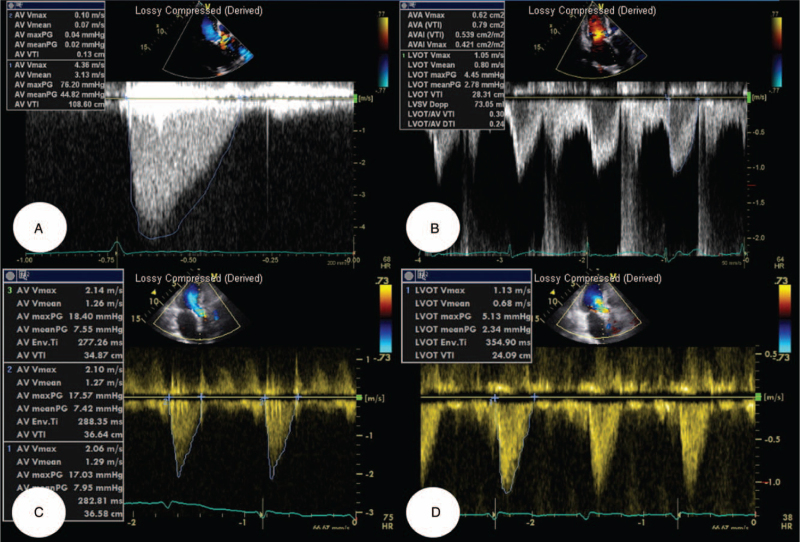
(A-B) Before TAVR, transthoracic echocardiogram was performed, demonstrating a peak velocity was 4.36 m/s, and a mean pressure was 44.8 mmHg in AoV, suggestive of severe AS. (C-D) After successful transsubclavian TAVR, a peak velocity was dropped to 2.06–2.14 m/s, with a reduction of mean pressure to 7.42–7.95 mm Hg. AoV = aortic valve; AS = aortic stenosis; TAVR = transcatheter aortic valve replacement.

A 90-year-old Korean woman was brought to our department with dyspnea. Her medical history included chronic kidney disease, diabetes mellitus, hypertension, and ischemic cardiomyopathy. The patient previously underwent stent implantation in the right coronary artery (RCA) and left circumflex coronary artery (LCX). As her dyspnea symptoms gradually deteriorated, she visited our cardiovascular center for diagnosis and management. Her vital signs were as follows: temperature, 36.4°C, heart rate, 72 beats/min; respiratory rate, 20 breaths/min; and blood pressure, 140/80 mm Hg. On physical examination, grade IV systolic ejection murmurs were auscultated in both the upper and left lower sternal borders. Crackle sounds were heard in both chest areas, indicative of pulmonary edema. Chest radiography showed an increased cardiothoracic ratio and haziness in the right lower lung field. On the initial electrocardiogram, normal sinus rhythm was found. In the laboratory test, the high-sensitivity troponin-I was 0.047 ng/mL (reference range, 0–0.05 ng/mL) and the N-terminal pro-brain natriuretic peptide concentration was 5,043 pg/mL (reference range, 0–125 pg/mL). Because acute decompensated heart failure due to valvular heart disease was clinically suspected, 2-dimensional transthoracic echocardiography (TTE) was performed. In TTE, the peak velocity was 4.36 m/s, and the mean pressure was 44.8 mm Hg (Fig. 1A–B). Considering the aforementioned clinical manifestations, she was diagnosed with severe symptomatic AS combined with acute decompensated heart failure. First, we treated the patient with the best medical therapy for heart failure.

After medical therapy, including oxygen supplementation and intravenous furosemide injections, the patient's clinical condition stabilized. A multidisciplinary heart team reviewed the patients’ medical conditions. The mortality risk according to the European System for Cardiac Operative Risk Evaluation II was 8.81% and the Society of Thoracic Surgeons Predictive Risk of Mortality was 17.522%. At the heart-team conference, they concluded that TAVR was the optimal treatment for her.

Therefore, the patient underwent a workup for TAVR, including coronary computed tomography (CT) angiography, abdomen-pelvis CT, and coronary angiography via the right femoral artery. Coronary angiography showed well-deployed drug-eluting stents in the LCX and RCA; however, diffuse stenoses were found throughout the entire part of left anterior descending coronary artery, distal portion of the LCX, and proximal to the middle portion of the RCA.Abdomen-pelvis CT demonstrated diffuse atherosclerotic change in abdominal aorta, superior mesenteric artery, splenic artery and both iliac arteries (Figure S1, Supplemental Digital Content). Since we concluded that a transfemoral approach was not feasible in the patient owing to the greater burden of atherosclerotic changes in her vasculature, we planned to perform TS-TAVR instead.

**Figure 2:**
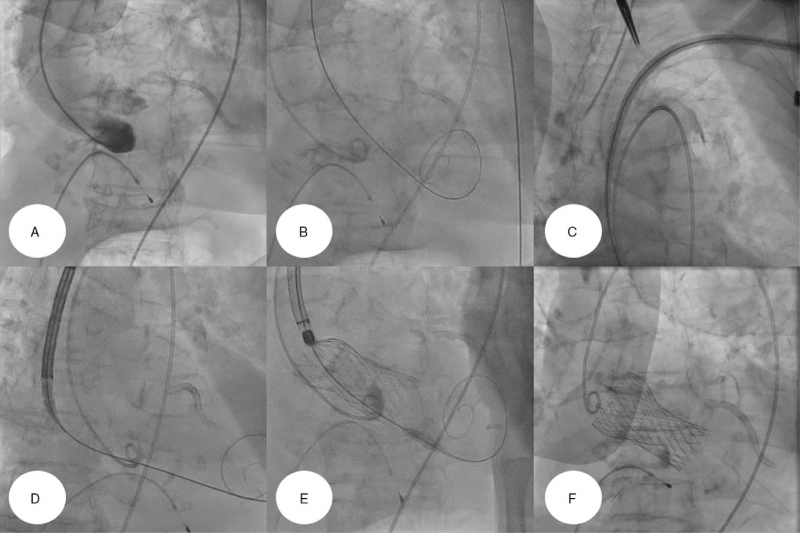
(A) We positioned a 5-Fr pigtail catheter around the ascending aorta, to perform an aortogram. (B) After a 0.035-inch Ampltz Super Stiff^TM^ guidewire (Boston Scientific Inc., Marlborough, MA) was introduced into the chamber of left ventricle, a Safari^TM^ guidewire (Boston Scientific Inc., Marlborough, MA) was also placed there using the catheter exchange technique. (C-D) After we inserted a 7-Fr vascular sheath into the subclavian artery, a 26 mm-sized self-expandable valve prosthesis (CoreValve^TM^ Evolut R^TM^, Medtronic Inc., Minneapolis, MN) was advanced through the vascular route of the left subclavian artery into the annulus of AoV. (E-F) Valve prosthesis was successfully deployed and expanded at the annulus of AoV. AoV = aortic valve.

TAVR was performed in a cardiac catheterization laboratory under general anesthesia. We initially introduced a 6-Fr pigtail catheter through the right femoral artery, placed it in the ascending aorta, and then performed an initial aortogram (Fig. 2A), showing that the ascending aorta was of sufficient length to perform TAVR. A temporary pacemaker wire was placed in the right ventricle via the right femoral vein (Fig. 2A). Thereafter, the proximal left axillary artery was exposed using a surgical incision and a 7-Fr sheath was inserted into the left subclavian artery. After a 0.035-inch Amplatz Super Stiff^TM^ guidewire (Boston Scientific Inc., Marlborough, MA) and a Safari guidewire were introduced into the left ventricle (Fig. 2B), we introduced a 26-mm self-expandable valve prosthesis (CoreValve^TM^ Evolut R^TM^, Medtronic Inc., Minneapolis, MN) through the vascular access of the left subclavian artery and successfully deployed it at the AoV annulus (Fig. 2C-F, Videos S1–S3, Supplemental Digital Content).

Afterward, the patient was transferred to the intensive care unit for close hemodynamic monitoring and was administered optimal medical therapy, including antiplatelet therapy (aspirin and clopidogrel), statin (simvastatin, 10 mg/day), beta-blocker (carvedilol, 12.5 mg/day) angiotensin receptor blocker II (olmesartan, 40 mg/day), calcium channel blocker (amlodipine, 10 mg/day), and diuretics (hydrochlorothiazide, 12.5 mg/day). The patient also received an intravenous furosemide injection to mitigate pulmonary edema and reduce oxygen demand. The patient was successfully discharged from mechanical ventilation several days later. Two weeks after hospitalization to the intensive care unit, the patient was successfully transferred to a general ward. Post-TAVR TTE showed a well-functioning valve with trivial paravalvular leakage, a decline in both peak velocity to 2.06–2.14 m/s, and a mean pressure gradient to 7.42–7.95 mm Hg. The area of the AoV was increased to 1.12 cm^2^ (Fig. 1C-D). Her dyspnea symptoms improved dramatically, and she was successfully discharged from our hospital.

## 3 Discussion

The present case demonstrates successful TS-TAVR in a nonagenarian female patient. The patient had a high burden of comorbidities, such as chronic kidney disease, diabetes mellitus, ischemic cardiomyopathy, and a history of percutaneous coronary intervention. In addition, the patient had clinical manifestations suggestive of acute decompensated heart failure at the time of admission, which required appropriate and urgent medical interventions. Because we judged that the femoral approach to this patient was infeasible owing to the greater burden of atherosclerotic change in her vasculature, TS-TAVR was performed.

As TAVR has been shown to improve quality of life, exercise capacity, and long-term clinical outcomes in patients with AS, it is well known as a minimally invasive treatment strategy applicable to elderly patients.^[12]^ Nevertheless, it seems risky in a super-aged population such as nonagenarian patients. According to a published study on transfemoral TAVR in the nonagenarian population, 30-day mortality in nonagenarians was approximately twice that in patients aged <90 years.^[12]^ However, some clinical data contradict this view.^[13,14]^ Stehli et al demonstrated that there was no significant difference in in-hospital complications and 30-day and 1-year outcomes between nonagenarian and non-nonagenarian groups.^[14]^ This result suggests that TAVR is a relatively feasible and safe procedure, even in a super-aged population.

Interestingly, the majority of clinical studies and case reports on TAVR in nonagenarians have focused on patients undergoing TAVR with a transfemoral approach.^[12–15]^ In a PubMed search for cases of TAVR in nonagenarian patients, 5 cases were reported in 5 articles (Table S1, Supplemental Digital Content).^[16–20]^ Among these cases, 4 patients were female, but 4 patients received the femoral approach during the TAVR procedure. No subclavian approach was used in these patients. In some comparative studies, an extremely small number of patients undergoing TS-TAVR have been identified.^[13,15]^ In other words, there have been no reported cases with a detailed description of TS-TAVR in nonagenarian patients. To the best of our knowledge, this is the first reported case of TS-TAVR performed in a nonagenarian patient with severe AS.

## 4 Conclusion

Here, we report a rare case of successful TS-TAVR in a nonagenarian patient with severe AS. We cautiously suggest that the subclavian approach may be feasible and safe as a femoral approach for TAVR in super-aged patients.

## Author contributions

**Data curation:** Seok Oh, Dae Young Hyun, Ju Han Kim, Kyo Sun Lee.

**Methodology:** Seok Oh, Dae Young Hyun, Ju Han Kim, Kyo Sun Lee.

**Writing – original draft:** Seok Oh.

**Writing – review & editing:** Ju Han Kim, Dae Young Hyun, Kyung Hoon Cho, Min Chul Kim, Doo Sun Sim, Young Joon Hong, Youngkeun Ahn, Myung Ho Jeong, Yochun Jung, Kyo Sun Lee.

## Supplementary Material

Supplemental Digital Content

## Supplementary Material

Supplemental Digital Content

## Supplementary Material

Supplemental Digital Content

## Supplementary Material

Supplemental Digital Content

## Supplementary Material

Supplemental Digital Content
