# Supplementary figures and images for: Successful subclavian transcatheter aortic valve replacement in a nonagenarian patient: Case report and review of literature
Source: Medicine (Baltimore). 2022 Jan 28;101(4):e28702. doi: 10.1097/MD.0000000000028702 (PMC8797506; doi:10.1097/MD.0000000000028702)

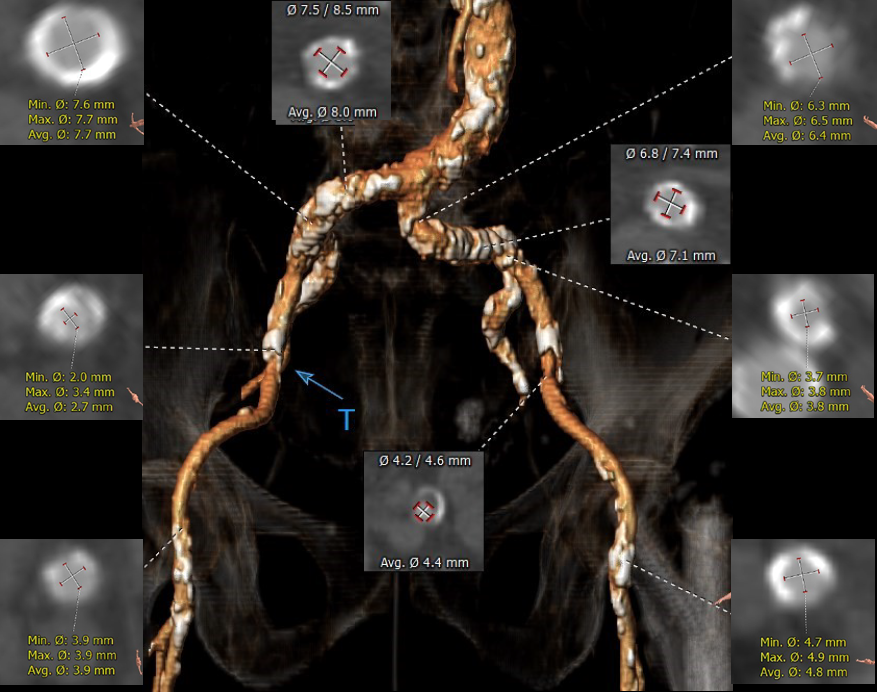

Supplement: Supplemental Digital Content [file medi-101-e28702-s001.tif]
