# Supplementary material for: Successful subclavian transcatheter aortic valve replacement in a nonagenarian patient: Case report and review of literature
Source: Medicine (Baltimore). 2022 Jan 28;101(4):e28702. doi: 10.1097/MD.0000000000028702 (PMC8797506; doi:10.1097/MD.0000000000028702)
Supplement: Supplemental Digital Content [file medi-101-e28702-s002.docx]

**Supplemental Table 1.** Clinical characteristics of published cases about TAVR in nonagenarian patients

| Number | Author | Year | Number of Cases | Sex | Age | NYHA Grade | Comorbidities | PCI | Vascular Approach |
| --- | --- | --- | --- | --- | --- | --- | --- | --- | --- |
| 1 | Fennelly | 2018 | 1 | Female | 90 | III | HTN, DL, AF, stroke, PVD, and AAA | None | Femoral |
| 2 | Kneitz | 2013 | 1 | Female | 95 | Not described | HTN, COPD, hypothyroidism, congestive heart failure, AF, CKD | None | Not described |
| 3 | Matsuo | 2017 | 1 | Female | 90 | II | CKD | None | Femoral |
| 4 | Mouton | 2019 | 1 | Female | 93 | Not described | CAD, AF, autoimmune factor V deficiency | None | Femoral |
| 5 | Zegrean | 2017 | 1 | Male | 97 | III | None | None | Femoral |

AAA = abdominal aortic aneurysm; AF = atrial fibrillation; CAD = coronary artery disease; CKD = chronic kidney disease; DL = dyslipidemia; HTN = hypertension; PCI = percutaneous coronary intervention; PVD = peripheral vascular disease; TAVR = transcatheter aortic valve replacement.
